# Non-Conventional Clinical Uses of TSH Receptor Antibodies: The Case of Chronic Autoimmune Thyroiditis

**DOI:** 10.3389/fendo.2021.769084

**Published:** 2021-11-05

**Authors:** Giorgio Napolitano, Ines Bucci, Giulia Di Dalmazi, Cesidio Giuliani

**Affiliations:** ^1^ Department of Medicine and Sciences of Aging, Unit of Endocrinology, University “G. d’Annunzio” of Chieti-Pescara, Chieti, Italy; ^2^ Center for Advanced Studies and Technology (CAST), University “G. d’Annunzio” of Chieti‐Pescara, Chieti, Italy

**Keywords:** chronic autoimmune thyroiditis, TSH-receptor blocking antibodies, TSH-receptor stimulating antibodies, Hashimoto’s thyroiditis (HT), atrophic thyroiditis

## Abstract

Anti TSH receptor antibodies (TSHrAb) are a family of antibodies with different activity, some of them stimulating thyroid function (TSAb), others with blocking properties (TBAb), it is a common finding that antibodies with different function might coexist in the same patient and can modulate the function of the thyroid. However, most of the labs routinely detect all antibodies binding to the TSH receptor (TRAb, i.e. TSH-receptor antibodies detected by binding assay without definition of functional property). Classical use of TSHr-Ab assay is in Graves’ disease where they are tested for diagnostic and prognostic issues; however, they can be used in specific settings of chronic autoimmune thyroiditis (CAT) as well. Aim of the present paper is to highlight these conditions where detection of TSHr-Ab can be of clinical relevance. Prevalence of TSHrAb is different in in the 2 main form of CAT, i.e. classical Hashimoto’s thyroiditis and in atrophic thyroiditis, where TBAb play a major role. Simultaneous presence of both TSAb and TBAb in the serum of the same patient might have clinical implication and cause the shift from hyperthyroidism to hypothyroidism and vice versa. Evaluation of TRAb is recommended in case of patients with Thyroid Associated Orbitopathy not associated with hyperthyroidism. At present, however, the most relevant recommendation for the use of TRAb assay is in patients with CAT secondary to a known agent; in particular, after treatment with alemtuzumab for multiple sclerosis. In conclusion, the routine use of anti-TSH receptor antibodies (either TRAb or TSAb/TBAb) assay cannot be suggested at the present for diagnosis/follow up of patients affected by CAT; there are, however, several conditions where their detection can be clinically relevant.

## Introduction

Hashimoto’s thyroiditis is a chronic autoimmune disorder that has been described for the first time by Hakaru Hashimoto in 1912 (1). His description: “*a massive growth of lymphatic elements, primarily lymphoid follicles” …. “this condition was a ‘destructive affectation of the thyroid … here and there infiltrated with clumps of cells … which are found to be composed of leucocytes*” depicts the spectrum of the chronic lymphocytic thyroiditis which is the most common type of chronic autoimmune thyroiditis (CAT). Nowadays the term Hashimoto’s thyroiditis (HT) is commonly and erroneously used to identify all forms of CAT and is considered the most common cause of hypothyroidism as well as the most common autoimmune and endocrine disease (2). However at least one different form of disease is classified within the CAT, i.e. the atrophic thyroiditis (AT). In 1873 Sir William Withney Gull in his seminal study “On a cretinoid state supervening in adult life in women” illustrated the following picture: “*Her face altering from oval to round … the tongue broad and thick, voice guttural, and the pronunciation as if the tongue were too large for the mouth (cretinoid)… In the cretinoid condition in adults which I have seen, the thyroid was not enlarged … at a first hasty glance there might be supposed to be a general slight oedema of it.*” (3) which is considered the first description of the atrophic form of CAT.

Distinction of the two main forms of CAT has not relevance only for a proper categorization of the disease, but also because the role of the anti TSH receptor antibodies (TSHrAb) is various and has a different weight in the two forms. Recognizing the 2 forms might be difficult, the most distinctive clinical feature being thyroid atrophy which appears at the clinical onset in AT while it appears after a long-standing disease in HT. Of course, it might be difficult to notice this temporal distinction in clinical practice.

The first evidence for a serum thyroid-stimulating factor has been described in 1956 by Adams and Purves (4) and in 1958 by Mc Kenzie (5). Much work has been done from then, particularly in the last 2-3 decades; it is a major improvement that measurement of TSHrAb is suggested in the management of Graves’ disease according to the 2016 American Thyroid Association (6) while only in the 2011 version it was recommended TSHrAb measurement only as “an alternative means to diagnose GD”, to be used when a thyroid scan and uptake is unavailable or contraindicated (7). Moreover, in the “2019 European Thyroid Association guidelines on the Management of Thyroid Dysfunction following Immune Reconstitution Therapy” (8) TSHrAb assay is considered a cornerstone both of diagnosis and follow up. After the cloning and sequencing of the TSH receptor (TSHr) (9–11) we now know that TSHrAb are a family of antibodies with different activity, some of them stimulating thyroid function (TSAb), others with blocking properties (TBAb) and finally some neutral, i.e. with no effect on TSH binding and no effect on cAMP levels [TBII (12–14)], which are also called “cleavage” Abs because they recognize linear epitopes within the hinge region of the TSH receptor and might be involved in several signalling processes also including thyroid cell apoptosis (15, 16). For the purposes of this review, this last family of Abs have a minimal role. It is also of little clinical interest to know if a proportion of TBAb are indeed “weak” TSAb and their blocking activity is only due to preventing the binding of more potent TSH receptor agonist, like TSH or “potent” TSAb, as suggested by some Authors (17); from a clinical point of view they exert a blocking effect.

However, a major problem in interpretation of the role of TSHrAb arises from the observation that most of the labs routinely detect all antibodies binding to the TSH receptor without distinction of the functional properties.

**Figure 1:**
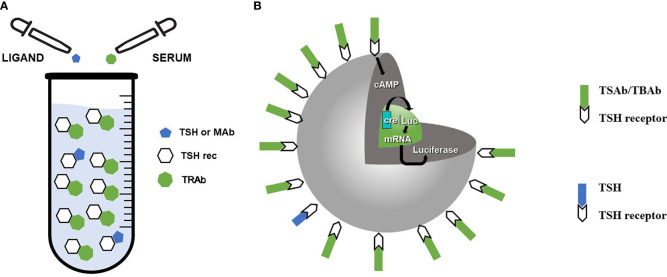
Different methods of TSHrAb assay. **(A)** Assays employing competition by TRAb for binding of a ligand (TSH or monoclonal antibody) to the TSHR. **(B)** Bioassays involving cultured thyroid cells or nonthyroidal cells expressing the TSH receptor. Binding of TSAb or TBAb modulate cAMP production. In the depicted assay cell lines lacking TSHR are double transfected with a luciferase reporter gene under the transcriptional control of cAMP-responsive elements (CRE-Luc) and the TSH receptor. TSAb/TBAb levels in patient sera are determined by measuring the production of luciferase.

Indeed, two types of assays have been historically used in the clinical setting: assays only measuring TSHrAb binding to the TSHr without functional discrimination (usually termed TRAb) and bioassays measuring functional activity of TSHrAb (12–14) (**Figure 1**). While the former, during decades, have been based on the inhibition by TSHR antibodies of radiolabeled/fluorescent TSH binding to thyroid membranes, human TSHr or known monoclonal human TSHR autoantibody, the latter measures the activation of transduction pathways after the binding of a ligand to the TSHr (17, 18). A further clarification is needed for the functional assay: it can reflect a net sum of TSAb and TBAb activities (and therefore give an estimate of the stimulus present on thyroid cell at the time of the blood test) or differentiate (by using different cells or protocols) between TSAb or TBAb activities (12, 19). Differently from Thyroglobulin antibodies (TgAb) and Thryroid peroxidase antibodies (TPOAb), which have no major role in the pathogenesis of autoimmune thyroid diseases, TSHrAbs are directly causative of Graves’ disease and are also involved in the pathogenesis of CAT. It is therefore obvious that an assay which gives a quantitative measure of the stimulus/inhibition performed on thyroid cells has more clinical advantages than an assay just measuring the Abs binding to the TSH receptor. Indeed, bioassay have clinical advantages in Graves’ disease because they have prognostic value (20) and are helpful and predictive in Graves’ patients during pregnancy/postpartum, as well as for extrathyroidal manifestations (21). It is beyond the aims of the present review to discuss advantages/disadvantages of the two different types of assays; a recent review (18) is exhaustively summarizing this issue. It is fair to admit, however, that binding assays are easier to handle also because commercially automated assays are available, and this is probably the main reason for their predominance (12–14, 18).

While the presence of TSAb in Graves patient is clearly causative and the amount of TSAb is correlated to the clinical entity of the disease, the role of TSHrAb (both TSAb and TBAB) in CAT is less defined. Marked lymphocytic infiltration (mainly T lymphocytes) of the thyroid is the hallmark of Hashimoto’s thyroiditis and apoptotic destruction and cell death of thyroid cells are probably the main mechanisms of damage leading to hypothyroidism. A minor contribution can also be attributed to TPOAb and TgAb which can be able to fix the complement and induce antibody-mediated complement-dependent cytotoxicity (ACDC) [revised in (18)]. A role for TSHrAb, and especially for TBAb which can block the TSH receptor and therefore inhibit both thyroid hormone synthesis and production and cell growth has been proposed especially for the atrophic form of thyroiditis. More speculative, as discussed later, is the role of TSAb.

## Clinical Uses of TSHr Ab in CAT

Aiming to better understand the role of TSHr Ab in chronic autoimmune thyroiditis, however, the impossibility to know in many studies whether TSAb or TBAb are involved, because binding assays have been used, it’s a major limit. It is a common finding that antibodies with different function might coexist in the same patient and can modulate the function of the thyroid (15–18, 22, 23). The final evidence for the contemporaneous presence of both kind of Abs has been obtained by the cloning of a stimulating (K1-18) and a blocking (K1-70) monoclonal Ab in the serum of the same patient (24).

According to an extensive review about the role of TSH receptor in clinical settings published in the year 2000 (14), mean prevalence of TRAb varies was 9% in HT and 12% in AT. However, when TBAb are separately evaluated mean prevalence was 12% and 33% in HT and AT respectively (**Table 1**). In a study by Cho et al. (25) TRAb were detected in 6.3% and in 48% and TBAb in 10.5% and 59% in goitrous and atrophic form respectively. More recent data have shown 9% and 25% TBAb positivity in adults patients with HT and AT, respectively (26), and a positive rate for TBAb of 38% in children with AT (27). Finally, a study from Kahaly (28), evaluating patients with autoimmune thyroiditis without distinguishing between the 2 two main subgroups, showed TRAb positivity in 9.4% of sera and TBAb positivity in 9.3%; interestingly TSAb and TBAb positivity has been registered in 6/67 sera, thus meaning that contemporaneous presence of both TSAb and TBAb can be found not only in Graves’ disease but also in CAT.

**Table 1:** Nomenclature of TSH-receptor antibodies and Prevalence of TRAb and TBAb in Hashimoto’s thyroiditis and atrophic thyroiditis according to previous studies.

Nomenclature			Definition			
TSHr-Ab			All TSH receptor antibodies without definition of assay method or functional properties			
TRAb			TSH-receptor antibodies detected by binding assay without definition of functional property			
TSAb			TSH-receptor stimulating antibodies which act as agonists by stimulating thyroid growth and thyroid hormone synthesis			
TBAb			TSH receptor blocking antibodies which act as antagonists, by blocking the action of the TSH			
	**Orgiazzi J et al.** (14)		**Cho BJ et al.** (25)		**Takasu N et al.** (26)	
	HT	AT	HT	AT	HT	AT
TRAb % (range)	9 (0-44)	12 (0-54)	6.3	48	–	–
TBAb % (range)	12 (0-44)	33 (0-62)	10.5	59	9	25

HT, Hashimoto’s thyroiditis; AT, atrophic thyroiditis.

Hashitoxicosis is usually referred to a transient phase of hyperthyroidism in patients with chronic autoimmune thyroiditis; it is usually attributed to destruction of thyroid cells and the release of stored hormones within the bloodstream. However, in 1971 Fatourechi et al. (29) described patients with hyperthyroidism indistinguishable from Graves’ disease (including elevated radioiodine uptake and TRAb positivity) but with histological features of Hashimoto’s thyroiditis; the hyperthyroidism was lasting few months and the patients later became hypothyroid. It has been therefore supposed that hyperthyroidism was attributable to the presence of TSAb. From then it has been generally accepted that a small percentage of hyperthyroid presentation of chronic autoimmune thyroiditis is due to the presence of TSAb. Contemporaneous presence of both TSAb and TBAb has been advocated as a mechanism by which euthyroidism can be maintained in a small subset of patients (28). Presence of TSAb during Hashimoto’s thyroiditis has also relevance in newborns and in pediatric age. Indeed, thyrotoxicosis in a newborn from a mother with chronic autoimmune thyroiditis treated with L-T4 has been described (30); the presence of TSAb has been documented and it was the cause of hyperthyroidism lasting only until TSAb normalized. Transient central hypothyroidism, possibly due to TSH suppression, then developed and the newborn required L-T4 treatment.

TBAb have a clearer role in CAT; as previously briefly disclosed, TBAb are supposed to have a central role in the etiology of the atrophic form of chronic autoimmune thyroiditis. They inhibit the metabolic pathways activated by the interaction of TSH with its receptor and then prevent both growth and hormone synthesis. It is therefore easily comprehensible that their presence is associated to a more severe form of hypothyroidism, especially in children (28, 31). In a population of patients with subclinical hypothyroidism, overt hypothyroidism and atrophic thyroiditis TBAb positivity has been detected in 12.5, 23.3 and 34% of sera, respectively (32). The same group of Authors, by using a different assay, also reported TBAb positivity in 46, 3, 9.4 and 36% of sera from patients with atrophic thyroiditis, euthyroid, subclinical or overt hypothyroid Hashimoto’s thyroiditis, respectively (33). In pediatric population presence of TBAb may be even more relevant, if 9% of children with CAT had TBAb posititvity which was corelated to higher values of TSH (31, 34). It is surprisingly interesting, however, to note that TBAb are also linked to a transient form of CAT, with spontaneous remission of hypothyroidism. Indeed, in a 11-years follow up in sera from Hashimoto’s thyroiditis the disappearance (35) of TBAb was linked to recovery of euthyroidism in about 40% of patients; it was only detected in patients with the goitrous form of CAT, while all the patients showing features of the atrophic form of thyroiditis remain hypothyroid. It is therefore accepted that in a small amount of patients with CAT and positivity for TRAb a spontaneous recovery from hypothyroidism can occur (36) and that TBAb in this small subset of patients are causative of the transient hypothyroidism (37). Of course, the small number of patients occurring this eventuality does not grant systematic assay of TBAb in all patients with Hashimoto’s thyroiditis. Transient positivity of TBAb has also relevance in congenital hypothyroidism. In 1980 Matsuura et al. described the first report of neonatal transient hypothyroidism correlated to the presence of maternal TBAb (38). Prevalence of congenital hypothyroidism due to the presence of TBAb is supposed to be approximately 1:180000 newborns (36) and reviewed in (39).

An intriguing, although infrequent phenomenon is the shift from hypothyroidism to hyperthyroidism and vice versa. It is supposed to be, at least partially, correlated to a shift of TBAb to TSAb or vice versa and has been matter of an extensive review (17). The evolution from hyperthyroidism to hypothyroidism is somehow a more frequent phenomenon which can be attributed to different causes; contemporaneous presence of Graves’ disease and Hashimoto’s thyroiditis is not difficult to understand, whether we keep in mind that these are the two far ends of the same disease called thyroid autoimmunity. It is therefore conceivable to imagine that, while TSAb stimulate thyrocytes to overwork, slower mechanisms of thyroid destruction can take place and with time prevail, especially when amount of TSAb decreases either spontaneously or due to anti-thyroid drugs (ATD). Shift from TSAb to TBAb can therefore be only a minority phenomenon in the evolution from hyperthyroidism to hypothyroidism. The opposite (i.e. the evolution from hypothyroidism to hyperthyroidism) is a very sporadic phenomenon which requires the development of TSAb acting on an already damaged thyroid or the shift from TBAb to TSAb. Both mechanisms have been described; in 2012 (26) Takasu and Matsushita reported the development of TSAb positivity in 2/34 (5.9%) of patients with TBAb positivity over a 10 years follow up. On the opposite few patients developed hyperthyroidism following the appearance of TSAb (40, 41) [and reviewed in (17)] without a previous TBAb presence; this phenomenon was accompanied by an increase of TgAb and TPOAb titer. There is no difference in age or sex ratio, most of the patients have been described in Japan, possibly due to the more common use of TRAb assay. The common feature of all patients developing TSAb is the long time, up to 20 years (41), treatment with L-T4. A possible link to TSAb development is difficult to imagine; a decrease of TBAb amount similarly to what happen for TgAb and TPOAb in long-time L-T4 treated patients (42), can be supposed but it remains difficult to understand why TSAb should behave oppositely to TBAb. Easier to understand is the phenomenon by which a newborn from a mother with chronic autoimmune thyroiditis should initially be hypothyroid and then develop hyperthyroidism. In 1983 Zakarjia et al. (43) described delayed onset of hyperthyroidism in a newborn from a mother with both TBAb and TSAb. The phenomenon has been attributed to the earlier disappearance of TBAb from the serum with the longer lasting presence of TSAb causing hyperthyroidism.

Thyroid Associated Orbitopathy (TAO) generally occurs in patients with hyperthyroidism due to Graves’ disease, and it sometimes occurs in euthyroid and hypothyroid patients. Mild orbitopathy, mainly consisting in mild upper eyelid retraction, has been reported to be common in chronic autoimmune thyroiditis (44), while severe ophthalmopathy was considered very infrequent. However, a recent study (45) evaluating 700 patients with CAT revealed that 44 (6%) of them had TAO, and 15/44 (34% of this subset and 2.1% of the whole CAT population) had an active/severe disease. Interestingly 30/44 (68.2%) of patients with CAT and TAO had TSAb positive values, while only 36/656 (5,5%) of patients with CAT and no TAO were TSAb positive; even more significant, all the 15 patients with active/severe disease had positive TSAb values. Although cases of severe thyroid-associated orbitopathy have been documented in Hashimoto’s thyroiditis (46), the recently published Guidelines for the medical management of TAO (47) suggest the opportunity to assay TRAb in all patients with TAO and either Graves’ disease or Hashimoto’s thyroiditis for diagnostic and prognostic purposes.

In a very unusual scenario CAT is associated with encephalopathy and this association, later defined “steroid-responsive encephalopathy associated with autoimmune thyroiditis (SREAT)” (48) has been described for the first time in 1966 (49). A serological distinctive feature of the disease is the contemporary presence of one or more thyroid antibodies (TgAb, TPOAb and TRAb) and antibodies against brain-expressed autoantigens; amongst the latter anti-alpha-enolase antibodies are the most frequent (50). Interestingly a recent study (50) has shown homologies between TSH-R and alpha-enolase; keeping in mind that main thyroid antigens (Tg, TPO and TSHr) are also expressed in the central nervous system and that alpha-enolase is expressed also in the thyroid, a pathogenic contribution of TRAb might be speculated.

Secondary CAT is a quite new clinical entity where onset of CAT is secondary to a known agent; interferon alpha or beta, interleukin-2, thalidomide, amiodarone, radiation therapy, lithium, immune checkpoint inhibitors, and thyrosine kinases inhibitors are some examples (2, 8). Central to the topic of this review is the onset of thyroid autoimmunity caused by immune reconstitution therapy, i.e., the restoration of immune cells after a depletion phase. This is particularly the case for three different therapies, i.e. following alemtuzumab, after highly active antiretroviral therapy (HAART) and finally after bone marrow transplantation (BMT) or hematopoietic stem cell transplantation (HSCT) (8). Of particular interest is the case of alemtuzumab treatment in patients with multiple sclerosis (MS); indeed, the first peculiarity to be noted is that high prevalence of thyroid dysfunction has only been observed in patients affected by MS (51, 52) but not when alemtuzumab has been used for rheumatoid arthritis, B-cell chronic lymphocytic leukaemia or transplantation (53–55). Therefore, a common genetic background can be postulated. Mechanism of action of alemtuzumab is based on its binding to CD52 antigen which is expressed on almost all T and B lymphocytes; after binding a rapid cell lysis is induced and lymphocytes almost disappear from the bloodstream. Then a quicker recovery of B cells (3-12 months) is observed while CD8+ and CD4+ T lymphocytes recover later (within 20 and 36 months respectively) [reviewed in (56)]; this gap might explain the onset of B-cell related thyroid autoimmunity. Interestingly, no onset of autoimmune disorders has been observed after B-cell only depleting immunomodulatory agent, such as rituximab (57). Aside from mechanistic explanation, a huge number (34 to 41%) of patients treated with alemtuzumab for MS (8, 56, 58) develop thyroid autoimmunity, mainly hyperthyroidism (from 4,7 to 33%), but also hypothyroidism (5 to 13,8%) and destructive thyroiditis (4%) as well. These data are even more impressive if compared with those observed in MS patients treated with interferon-beta (6.5%). Two sets of data have particular relevance to the topic of these review: 1) 70% of patients developing thyroid dysfunction had positive TRAb values and this value rose up to 76.7% in patients with hypothyroidism, thus suggesting the presence of TBAb, which has been confirmed by following studies showing TBAb as the cause of hypothyroidism in 50% of MS patients (59). 2) there is a high possibility to observe a shift from hyperthyroidism to hypothyroidism and vice versa (19 to 52%) and this change very often (up to 70%) occurs when TRAb are present. For these reasons the European Thyroid Association in its guidelines (8) does not recommend TRAb assay before alemtuzumab treatment or during routine follow up, but strongly recommend its use when thyroid dysfunction is disclosed (recommendations 11 and 14). It is also suggested that TRAb assay might be performed in hypothyroid patients with a previous detection of Abs-positivity, because of the possible transitory presence of TBAb thus resulting in recovery from hypothyroidism.

## Concluding Remarks

The routine use of anti-TSH receptor antibodies (either TRAb or TSAb/TBAb) assay cannot be suggested at the present for diagnosis/follow up of patients affected by CAT. Indeed, prevalence of TRAb is too low in CAT to be a useful tool for diagnosis; moreover, even when detected they do not modify the strategy of treatment. There are, however, several conditions where their detection can be relevant: 1) due to the possibility of disappearance of TBAb which can be causative of transient hypothyroidism in goitrous form of CAT (35), HT patients with long standing stability of low dose L-T4 treatment might deserve evaluation of TBAb because of the high rate of recovery (40%) in patients whose hypothyroidism was originally due to the presence of TBAb. It has to be noted, however, that commercial test of TBAb is hard to find [revised in (18)]; 2) when a shift from hypothyroidism to hyperthyroidism is observed in a patient with long time treatment with L-T4; 3) in patients with alternating hyper/hypothyroidism phases; 4) in a newborn from a mother with CAT and a contradictory clinical setting; 5) in patients with CAT and suspected TAO. Assay of TSHrAB (either TRAb or, better, TSAb/TBAb) is, on the contrary, mandatory when treatment with immune reconstitution therapy (especially in MS patients) is performed and thyroid dysfunction is detected.

Finally, it must be noted that studies on the clinical role of TSHrAb in CAT are scarce and available data are very rarely based on studies on numerous population; therefore, assay of TSAb and particularly of TBAB should deserve more clinical studies to better understand their role.

## Authors Contributions

GN: substantial contributions to the conception and design of the work; reviewing the literature; drafting the work. IB: substantial contributions to the conception of the work; revising the work critically for important intellectual content. GD; substantial contributions to the design of the work; revising the work critically for important intellectual content. CG: substantial contributions to the design of the work; revising the work critically for important intellectual content. All authors contributed substantially to the article and approved the submitted version.

## Conflict of Interest

The authors declare that the research was conducted in the absence of any commercial or financial relationships that could be construed as a potential conflict of interest.

## Publisher’s Note

All claims expressed in this article are solely those of the authors and do not necessarily represent those of their affiliated organizations, or those of the publisher, the editors and the reviewers. Any product that may be evaluated in this article, or claim that may be made by its manufacturer, is not guaranteed or endorsed by the publisher.
